# Hahnemann’s Method of Individualizing

**Published:** 1885-07

**Authors:** 


### HAHNEMANN'S METHOD OF INDIVIDUALIZING.

Hahnemann, in a letter to E. Stapf, dated Leipsic, January 24th, 1814, says:

The fevers last fall and this winter differ very much from those prevailing last spring; they naturally, therefore, require another treatment. As we, the "pitied" homœopathists, are so devoid of science that we do not want to be ruled by mere names—"nervous fever," "hospital fever," "typhus," etc.—we cannot satisfy ourselves with contrived recipes laid down in books for such names. What an easy time of it such of our colleagues as are not infected with our heresy have—looking into their pocket-manuals!

Besides the conditions and medicines before mentioned, of which you are already aware, we cannot do without Arsenic in such conditions as the following, which are produced by it in its pathogenetic effects:

1, a continual thirst, wherein the patient only wets his lips and cannot drink much;

2, has cold hands and feet;

3, overestimates his strength—venturing to get up and out of bed and then sinking down to the floor;

4, when he is continually anxious to get from one bed into another;

5, does not know what to do with himself on account of anxiety—mostly in the third hour of the night;

6, in which case, when he closes his eyes (and even otherwise) he sees persons and events before his eyes—often of neither a fearful nor an anxious, but merely an imaginary character;

7, the patient is faint-hearted, timid, inclined to weep, fears death;

8, sudden spells of suffocation befall him, particularly in the evening, when lying down, with or without cough;

9, or he struggles with frequent sickness and squeamishness.

In such cases you will see wonders effected by a single globule of the decillionth; you may rest assured of it.

We add to this truly master-sketch of characteristics some parallels to particular symptoms above for the instruction of students, keeping in view typhoid fevers only:

1. No remedy exactly like it; *Lycop.*, every little swallow becomes disgusting; *Sulph.*, because water disturbs the stomach; *Nat. mur.*, it does not taste well; *Sambucus*, it is not pleasant (*Calcar.*): 3, *Apium virus*, *Nat. mur.*: 4, *Calcar. c.*, *Cina*, *Sepia*, *Cham.* (*Verat.*, *Merc.*, *Hyos*, *Bellad.*, *Rhus*): 5, *Kali carb.*: 6, *Calcarea carb.*, *Sambucus*: 7, *Rhus*, *Verat.*, *Bryon.*, *Coccul.*, *Acon.*: 8, *Phosphor.*, *Pulsat.*: 9, *Phosp.*, *Lycop.*, *Bryon.*, *Calcar.*—C. Hg. in *J. of M. M.*

